# Determination of Temporal Spawning Patterns and Hatching Time in Response to Temperature of Atlantic Bluefin Tuna (*Thunnus thynnus*) in the Western Mediterranean

**DOI:** 10.1371/journal.pone.0090691

**Published:** 2014-03-07

**Authors:** Ana Gordoa, Gustavo Carreras

**Affiliations:** Department of Marine Ecology, Centro de Estudios Avanzados de Blanes, Spanish National Research Council (CSIC), Blanes, Gerona, Spain; University of Bologna, Italy

## Abstract

This study analysed the temporal pattern of Atlantic bluefin tuna (ABFT) spawning in the Balearic spawning ground and examined its reproductive performance after years in captivity. Furthermore, ABFT hatching time at different on-site temperatures was determined for the first time. Spawning surveys were carried out in 4 spawning seasons (2009–2012) aboard tuna transport vessels. Three groups of spawners were monitored: a captive group transported to the spawning region and monitored throughout the four spawning seasons and two wild groups caught in 2009 and 2010 which were transferred to a monitoring transport cage immediately after being caught. Surface plankton samples were collected nightly, beginning immediately after the first purse seine catches were made and concluding after spawning was observed to have ended. All groups displayed the same spawning hours, restricted between 2:00–5:00 a.m. The captive group, as they got older, shifted towards the earliest hour, suggesting an age influence on reproductive time. The onset of spawning varied annually from the end of May to the beginning of June at temperatures around 19°C–20°C, ending by the second week of July. The peak of spawning was consistently around the summer solstice, June 15^th^–30^th^. The results showed the negative effect of unstable oceanographic conditions in the spawning process which might influence the annual reproductive success of ABFT. The influence of temperature on hatching time was higher than that observed in other tuna species, twice as fast at 26°C (23 h) as at 19.5°C (49 h). Overall, this study shows the strength of the internal mechanism in ABFT that controls spawning traits. Spawning in ABFT is cyclical and highly synchronised on diel and annual scales. We consider that the timing of spawning is rather influenced by day length and its adaptive significance is discussed.

## Introduction

The Atlantic bluefin tuna (ABFT) is an emblematic species from every perspective: economic, biological, environmental and social. This is due to its high commercial value, its particular physiology and behavior and its wide spatial distribution over the entire North Atlantic and the Mediterranean Sea, where its fishery has roots that date back to 7000 BC [1]. The scientific effort dedicated to this species is remarkable, from its physiology [2], [3], migratory patterns [4]–[8], growth [9], feeding ecology [10]–[12] and reproductive biology [13]–[16], among others.

The ABFT is composed of two genetically different populations [17], the Western and the Eastern-Mediterranean, each with distinct spawning grounds, the Gulf of Mexico and the Mediterranean respectively, but both sharing feeding grounds on both sides of the Atlantic [4], [8]. The spawning migration towards the Mediterranean is seasonal and progressive: for five decades it has been known that fish enter through the Strait of Gibraltar in May-June and leave in July-August [18], with a staggered arrival at and exit from the spawning grounds. However, it is unknown whether spawning is likewise staggered, starting upon the arrival of the first spawners, or whether there is a waiting period until a particular set of required environmental conditions, such as temperature, become optimum, thus triggering spawning.

In the last decade, the acquired knowledge on the reproductive biology and reproduction of the Eastern population has been substantial. The spawning period and locations for ABFT in the Mediterranean Sea have been inferred from the spatial patterns of larval distribution [19]–[22], from histological findings [23], [24] and from electronic tagging [4], [25], [26]. The actual onset of spawning seems to be temperature-related, and 24°C was considered the threshold temperature for all tuna species [27]. However, temperatures reported in the ABFT spawning areas in the Mediterranean during the spawning period range from 21.5°C to 26.5°C [19], [21], [28], [29], but neither spawning nor water warming is simultaneous all over the Mediterranean. Temporary differences in spawning between Mediterranean regions are known: June–July in the western Mediterranean [16], [19], [21], [23], [24], [30]–[32], and one month earlier, May–June, in the Levantine Sea [33]. These findings were finally confirmed by a gonad maturation study across the Mediterranean [34] and are presumably related to the time elapsing before the threshold temperature is reached in each region. Therefore, all the current knowledge of the spawning period is based on and inferred from indirect valuations, and direct information on the ABFT spawning temporal dynamics remains lacking.

The influence of temperature on spawning dynamics and early life stage development was clearly observed in yellowfin tuna (*Thunnus albacares*) [35]. Moreover, the effect of temperature on egg time to hatching was modelled by Pauly & Pullin [36] for different pelagic species. The influence of temperature on the egg stage duration of ABFT is unknown but merits investigation, in particular in the Western Mediterranean. In this region the sea surface temperature rises rapidly during the ABFT spawning period (May–July), consequently an earlier (May) or later (July) spawning may have a direct impact on the hatching time and consequently on the extension of the exposed egg stage to predation, which might influence its survival success.

The direct observation of ABFT spawning in the natural system is a difficult task which is unlikely to be accomplished, and its long-term monitoring was unfeasible until tuna transport cages were shown to be effective spawning observatories. The ability of ABFT to regularly spawn in transport cages was observed and the nocturnal spawning could be determined [37]. Transport cages cannot be considered to present either pure captivity conditions like those specific to tuna farms, or wild natural conditions, as the fish are caged for transport and transfer to the farming facilities, but they are unique platforms for direct observation of spawning. Captivity categorisation might vary depending on the living conditions the spawners had before they were transferred to the transport cage. A group of wild spawners immediately transferred, after capture, to the transport cage are expected to behave more closely to wild spawners than those spawners kept in captivity for years.

This study has therefore attempted to assess the temporal spawning pattern of ABFT in the Western Mediterranean spawning grounds, around the Balearic archipelago, to expand the knowledge of the reproductive dynamics of this species, using transport cages as surveying elements. The study, originally planned for the 2009 spawning season, has been extended annually and new goals were progressively added until 2012. The specific objectives were: to determine the onset of spawning, to identify the peak spawning period, to determine the spawning duration, to analyse the variability of the spawning time, to analyse the natural reproductive capability of individuals held in captivity from 1 to 4 years when exposed to the same environmental conditions as wild spawners, and lastly to study the variability of the egg hatching time with on-site sea water temperature.

## Materials and Methods

The study was carried out with the cooperation of the Spanish company Balfegó Group, making use of the facilities provided by that company's fishing fleet including its tuna transport vessels and their catch. The transport of tuna groups under monitoring was done with the previous authorization from the Spanish Directorate-General for Fisheries. The study was carried out off shore where no specific permission is required. Hatching observations involved no harm to the eggs and no special permission was required for the development of the experimental activities.

**Figure 1:**
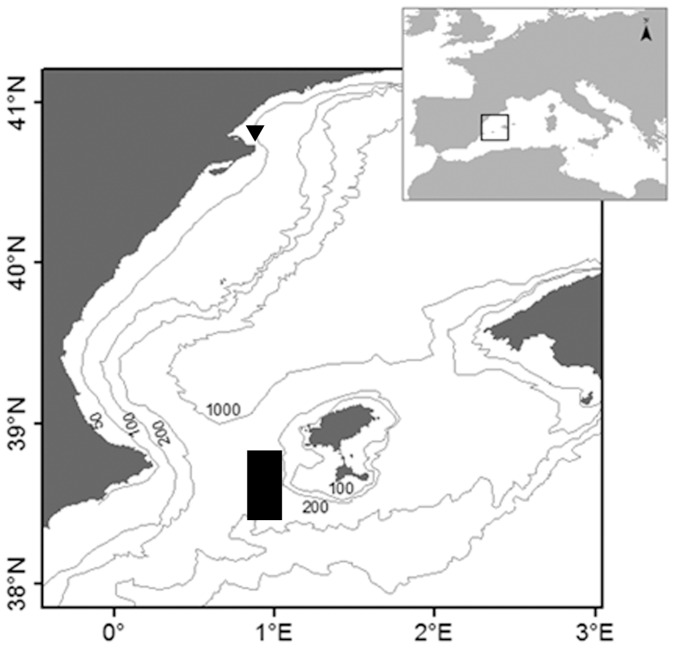
Map showing the primary location of the tuna transport cages during the spawning monitoring (█) and the location of the fattening facilities from where the captive groups was transported every spawning season (▴).

In 2009 the specific objectives were to identify the onset, peak and conclusion dates of spawning and to determine whether a year in captivity inhibited spawning. To accomplish the goals, two groups of tuna, a wild group (WG) and a captive group (CG) were monitored in two different transport cages. The WG was captured in the first purse-seine catches taken by the fleet in the Balearic spawning grounds and transferred to a transport cage for monitoring. Thus, the cage of the WG was filled with individuals from different schools. The CG, after one year in captivity, was transferred from the Balfegó Group's fattening facilities located off L'Ametlla de Mar, NE Spain, to a transport cage and moved to the spawning grounds, where it was held near the WG (Figure 1) to offset the possible effect of environmental conditions on the spawning process.

Sampling of both groups began immediately after the first catches were transferred. The CG was monitored until the end of June. In order to determine the duration of spawning of the WG, its monitoring continued until spawning was deemed to have definitely halted (Table 1). Spawning was considered to have ended when after a clear declining trend the plankton sampling nets did not collect any eggs on three consecutive days.

**Table 1:** Characteristics of the Atlantic Bluefin tuna groups under spawning monitoring and surveyed periods.

Year	Group	Total	Total	Individual	Density	Survey
		Number	Weight (kg)	weight (kg)	(kg/m^3^)	Period
2009	W	1190	146000	122	2.48	24^th^ May–21^st^ July
2009	C	1000	75000	75	1.27	24^th^ May–28^th^ June
2010	W	1025	130000	120	2.21	27^th^ May–16^th^ July
2010	C	835	65130	78	1.11	22^nd^ May–16^th^ July
2011	C	741	77064	104	1.31	23^rd^ May–18^th^ July
2012	C	659	85011	129	1.44	23^rd^ May–11^th^ July

Group's label correspond to those hold in captivity for more than a year (C) and to those caught immediately before the onset of the monitoring (W).

In 2010 the objectives were to add information on the annual variability of the onset date of spawning, to determine more precisely the spawning time and its changes during the spawning period, and to analyse the spawning behaviour after 2 years in captivity. The monitoring of WG began once it was caught by the very first purse-seine hauls (Table 1). In 2011 and 2012 the spawning was only monitored in the CG group to investigate the potential influence on spawning behaviour of the time held in captivity. This was achievable thanks to the agreement of the farming company in keeping the CG in a separate cage in the farming facilities, and every year, from 2009 to 2012, it was transported from the farm to the spawning area and back again to its cage when spawning was over.

The characteristics of the monitored groups are summarised in Table 1. The individual and total weight of the WG were visually estimated as these groups were transferred at sea from the purseiner's haul to the transport cage. The WG total numbers were estimated from the total weight divided by the individual weight. The CG was caught in 2008 and the initial number of individuals was counted during the transfer to the cage in the farming facilities. After 14 months in captivity, two months after the 2009 survey, the company begun to harvest some individuals, recording their length and weight for commercial and quality control reasons. Thus, the number of individuals of the CG group at the beginning of each survey was calculated by subtracting the sacrificed tuna over the period between surveys from the number of the previous survey. The average individual weight of CG was estimated from the annual average weight of the sacrificed tuna. It should be pointed out that the estimated average weight in 2009 was overestimated because tuna were only sacrificed after September. The cage dimensions were 50 m diameter by 30 m depth, the densities at which tuna are normally kept during the transport are those estimated for WG (Table 1). The tuna were fed with *Scomber japonicus* by an auxiliary vessel every four or five days during the surveys.

Egg sampling followed the plankton protocol for transport cages already tested successfully in an earlier study [37]. Samples were collected using bongo nets fitted with 0.3 mm-mesh nets deployed behind the transport cages at a depth of three metres. The towing speed of the transport vessels was constant at around 0.6 knots. The distance between the transport vessel and the rear of the cage was 200 m, and a rubber dinghy was used to transport and set the bongo nets at each station nightly. Sampling time (local time  =  UTC + 2 hours) was established around the time period, 3:00 a.m. to 4:00 a.m., in which spawning was consistently observed [37]. Three stations were sampled per night at 02:00-02:55 a.m., 03:00–03:55 a.m. and 04:00–04:55 a.m. Furthermore, exploratory stations were added when spawning was observed at the earliest or latest hour to avoid any potential miss of spawning. At each station surface water temperature was recorded. There is one single exception in the sampling time protocol, at the beginning of 2009 and before spawning was detected, when the night sampling was only carried out from 03:00–03:55 a.m.

Upon retrieval of the gear, plankton samples were immediately preserved in 5% buffered formalin. Semi-quantitative measurements of the egg volumes collected at each sampling station were estimated. The volume of eggs (in millilitres) in the samples collected at each station was estimated after settling in 250-ml translucent jars. At several stations 250-ml jars were too small to hold all the eggs collected, so the spare volume of eggs was measured before being returned to the sea or kept for hatching experiments.

The hatching experiments on board were carried out from samples taken in the 2010–2012 surveys. At several stations a fraction of live eggs (5 ml) was collected and immediately transferred to a 10 L container of 0.3 mm filtered seawater, after hatching they were immediately returned to the sea. The incubation container was placed in a shaded deck area exposed to ambient air temperature and indirect ambient light. To prevent heating, the seawater was changed twice a day. For each experiment the spawning time and temperature were recorded and temperature was monitored during the experiment until hatching. The experiments with a high degree of uncertainty of hatching time were disregarded, corresponding to those where the time elapsed between observed hatching and previous checking was longer than three hours. We investigated exponential and linear functions to model the influence of seawater temperature on ABFT egg hatching time.

For comparative purposes, the Pauly & Pullin [36] model for pelagic marine fish eggs in response to temperature and egg size was also fitted:
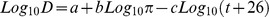
where D is days to hatching, π is egg diameter in mm and t is temperature in °C. In the particular case of *Thunnus thynnus* the egg size is around 1 mm [37], [38] so the model was reduced to temperature as the only explanatory variable. A total of 36 hatching experiments were carried out within an average temperature range between 20.5°C and 26°C.

## Results

During the four spawning seasons, the oceanographic conditions, and to a lesser extent technical problems, forced us to cancel some sampling days. The final number of successfully sampled stations was 1154.

**Figure 2:**
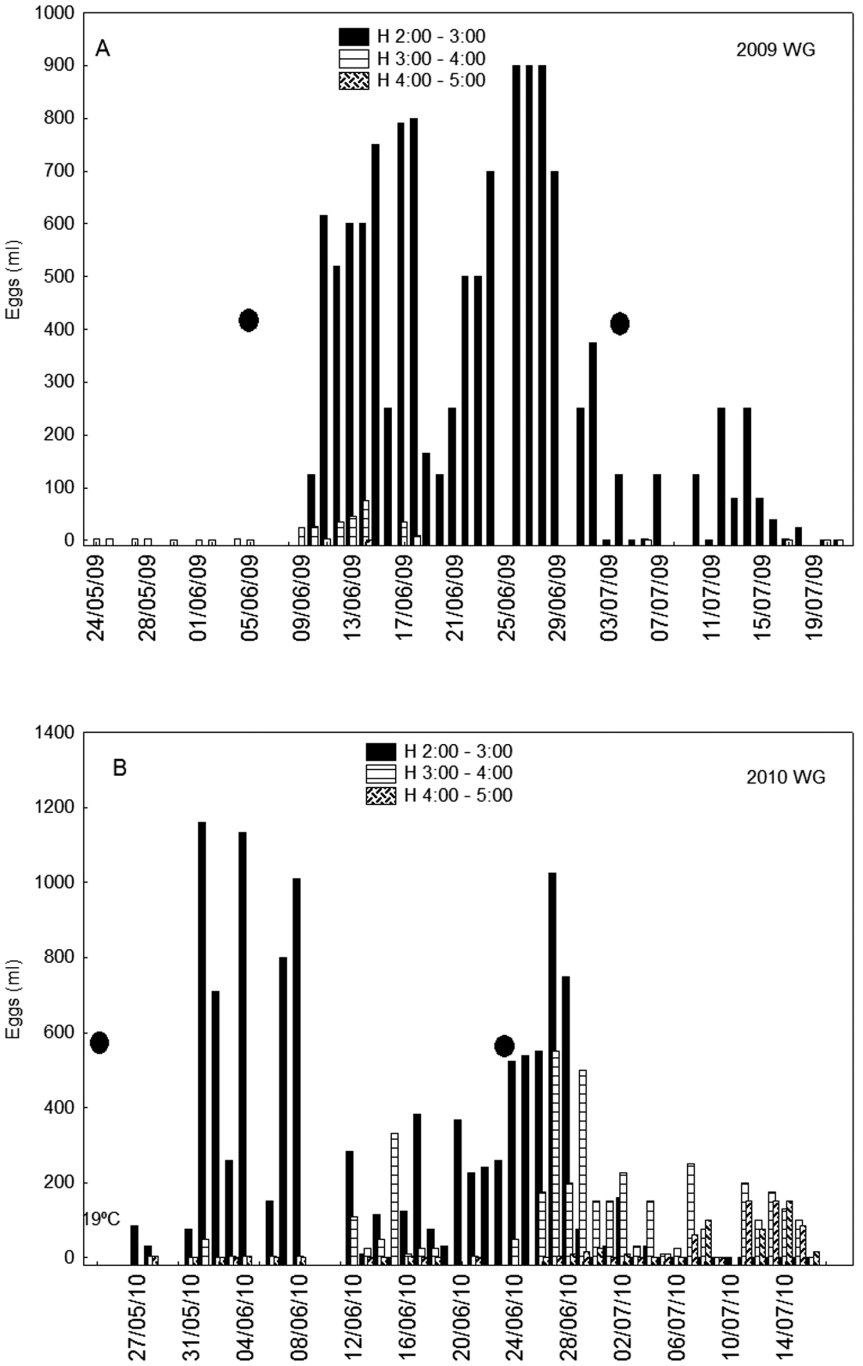
Volume (ml) of eggs collected per day and time interval from Atlantic bluefin tuna wild caged groups in 2009 (A) and 2010 (B) spawning surveys. (•) Full moon phase.

The WG in 2009, monitored from May 24^th^ to July 21^st^, showed the first signs of spawning on June 9^th^ (Figure 2A) with a sea surface temperature of 20°C. However, weather conditions prevented sampling on the three days preceding the appearance of the first eggs, so the possibility that spawning could have occurred between June 6^th^ and 8^th^ cannot be ruled out. The 2009 WG displayed a regular temporal spawning behaviour, adhering to the same peak spawning time, between 2:00 a.m. and 2:55 a.m., every night. Moreover, visual observations made from the rubber dinghy revealed that most spawning took place in the form of a short but intense burst, lasting for about 10 minutes between 2:00 and 2:15 a.m. This was the first recorded instance of visual sighting of wild ABFT spawning, and several days later an episode was caught on film as part of a documentary of Atlantic bluefin tuna [39].

The 2009 WG revealed intense spawning for approximately 21 days, from June 11^th^ to July 2^nd^, and the whole period of activity extended for around 38 days. Spawning slowed down after July 2^nd^ and ended by the 17^th^. Within the intense spawning period, a minimum took place at mid-term: this temporal pattern could be caused by the overlapping around June 20^th^ of two different time-shifted spawning groups, each with around 11 spawning days, but it can also be interpreted as a single spawning group, with a mid-term resting period. The latter hypothesis implies that the spawning pattern was synchronous for most of the group with peaks and resting periods and with a total spawning activity around 21 spawning days. This latter assumption is more consistent with the results obtained from other groups monitored in this study and with results from studies based on archival data discussed further in the next section.

The 2010 WG showed signs of spawning on the first sampled day, May 27^th^, with a sea surface temperature of 19.5°C (Figure 2B). Two aspects were distinctive from the previous year. Spawning was triggered earlier and at a lower SST, but in both years the beginning of spawning coincided with a date around a full moon. The spawning time was gradually delayed from 2:00–3:00 at the beginning of spawning to 4:00–5:00 at the end. The discontinuity of daily information due to the bad oceanographic conditions during the survey prevents inferences being made about separate spawning events. However, in view of the dates when spawning trends changed, the spawning from June 18^th^ to July 7^th^ could be a distinctive one, lasting around 19 days. But it may also result from two groups of spawners highly overlapped and with distinctive spawning times: 2:00–2:55 a.m. and 3:00–3:55 a.m.

**Figure 3:**
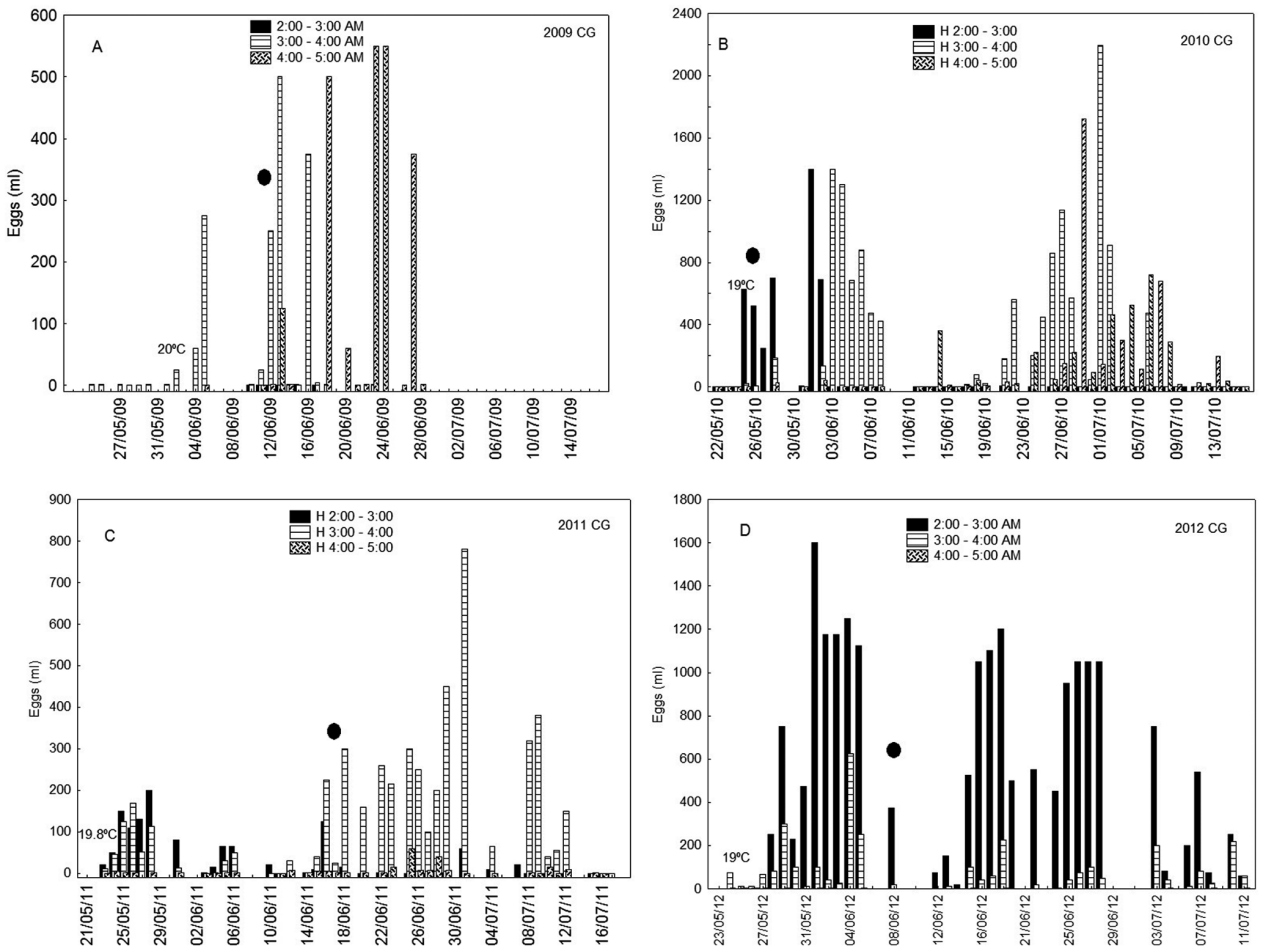
Volume (ml) of eggs collected per day and time interval from Atlantic bluefin tuna captive caged group in 2009 (A), 2010 (B), 2011 (C) and 2012 (D) spawning surveys. (•) Full moon phase.

The CG in 2009 was monitored for 35 days (May 24^th^–June 28^th^). The results clearly demonstrated that individuals held in captivity for one year were able to spawn (Figure 3A). In 2009, the CG began spawning one week earlier than the WG. Nevertheless, the temporal spawning behaviour of this group was irregular, as spawning did not take place on a daily basis and it gradually grew later as the season progressed, from 3:00–3:55 to 4:00–4:55. Although, at first sight, captivity could be inferred as the cause of the irregular spawning, later we had to reject it. The reproductive potential of this group in 2009 was presumably conditioned by the size/age of their individuals (Table 1), the most plausible reason as later results revealed.

The CG group in 2010, after two years in captivity, showed a regular daily spawning pattern (Figure 3B). The spawning began with an SST of 19°C, and two separate spawning periods could be depicted with peaks around June 4^th^ and 30^th^, respectively. Throughout the sampled period, the time of day when spawning occurred was gradually delayed from 2:00–3:00 at the beginning of spawning to 4:00–5:00 at the end. In 2011 in the CG, after three years of caging, the regular daily spawning was hidden by the bad oceanographic conditions and technical problems which prevented continuous monitoring. Spawning started on May 22^nd^ with an SST of 19.8°C (Figure 3C). The first spawning period could last 21 days, finishing around June 11^th^, and the second from June 14^th^ to July 4^th^. The gradual delay in the spawning time observed in the previous season was shortened; spawning around 4:00–4:55 was negligible. In 2012, one year later, spawning began practically on the same date, with a slightly cooler water temperature (19°C), and the time of spawning did not change through the sampling period, being concentrated between 2:00 and 2:55 a.m. (Figure 3d). As in previous years, the temporal pattern showed two separate spawning pulses in June and a smaller and less clear one in July.

**Figure 4:**
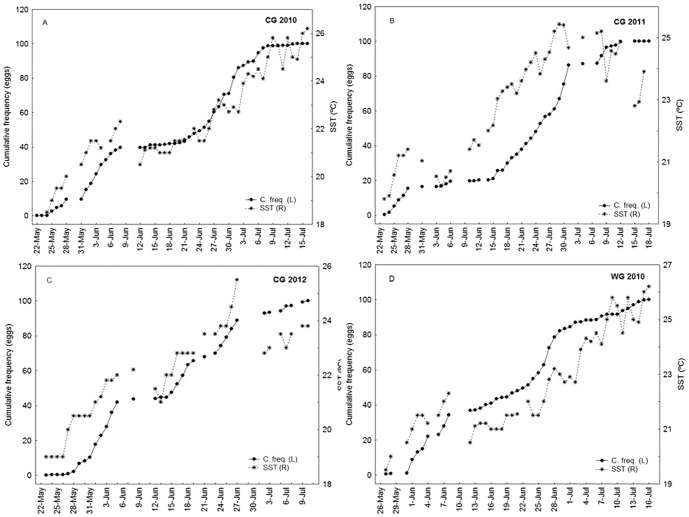
Cumulative percentage of spawning curve and water temperature.

Throughout these years of monitoring we observed that the onset of spawning coincide with a positive gradient in temperature (Figure 4). Additionally, we observed that once sampling was resumed, after several days of adverse oceanographic conditions, the first stations never showed spawning levels as intense as those observed before the storms, and frequently it stopped completely for several days. From the cumulative proportion of total daily eggs counted for each survey and daily SST, as an indicator of daily variability in oceanographic conditions, the cessation of spawning after adverse oceanographic conditions can be clearly depicted (Figure 4). The 2009 figures were excluded because the temperature sensors were damaged at the beginning of the survey. In 2010 the storm around June 9^th^ caused a sharp cooling of SST, a drop of 2°C, and both groups, the WG and CG, stopped spawning for several days: this break lasted longer in the CG. In 2011 and 2012 the storms were around the same dates, the end of May and beginning of June respectively, and the CG also stopped spawning for several days.

**Figure 5:**
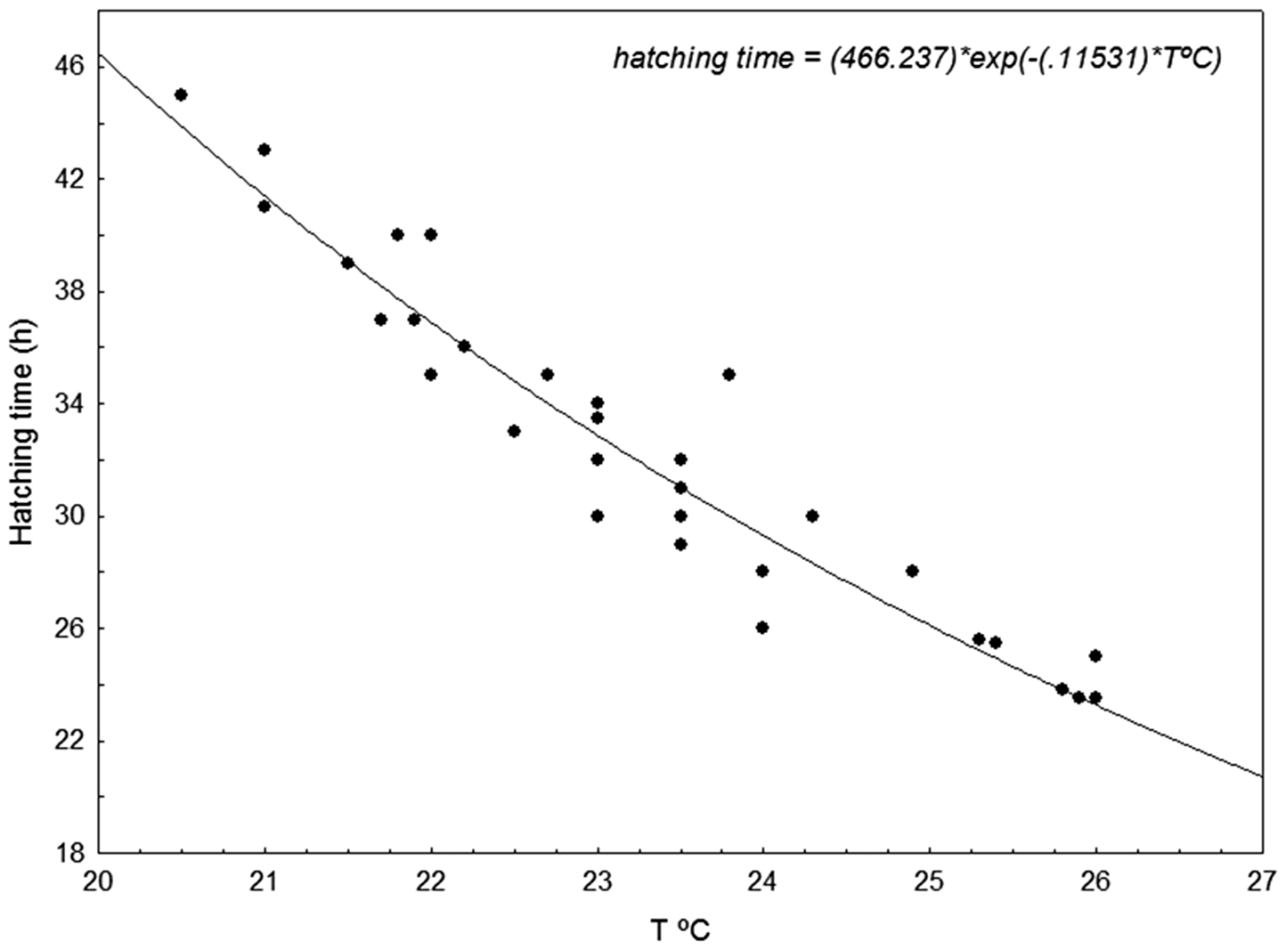
The negative exponential function showing the relationship between Atlantic bluefin tuna eggs hatching time and water temperature.

The results of hatching experiments revealed a significant role of temperature on the egg development rate (Figure 5). The mean water temperature in the egg incubations tanks ranged from 20.5°C to 26°C, and at the minimum temperature the hatching time (45 h) doubled the time elapsed at maximum temperature (23 h). The negative exponential function (Figure 5) was highly significant (P<0.0000), accounting for 90% of the observed variability.

The fitting to Pauly & Pullin's model [36] also explained 90% of hatching time variability.

Log_10_ D = 9.47−5.52 log_10_ (t+26). This result showed that temperature has a stronger effect (c = 5.52) for *Thunnus thynnus* than that estimated by Pauly & Pullin's model for pelagic marine fishes (c = 4.09).

## Discussion

This study reports the first direct observations on the temporal spawning patterns and hatching time of Atlantic bluefin tuna and reveals the capability of tuna transport cages as exceptional monitoring observatories. The results of this study are indicative of the strength of the internal mechanism in ABFT that controls spawning traits, which were undisrupted after years in captivity. We will discuss the potential sources of variability, extrinsic or intrinsic, observed on the spawning temporal dynamics of ABFT compared with those found for other tuna species.

The reproductive times have been attributed to be influenced by heritable variation, environmental effects, individual choice or a combination of all three [40] and also to phenotypic characteristic of spawners [41]. The three groups monitored in this study, two wild groups (WG) and the captive group (CG), displayed the same spawning hours, restricted between 2:00 and 5:00 a.m. This spawning time interval is shorter than those reported for other tuna species [35], [42], being the only one reported in full darkness. The spawning time of ABFT differs from those observed in bigeye tuna [43], yellowfin tuna [35], [44] and pacific northern bluefin tuna [42], which is indicative of a heritable species-specific component. Yet within the spawning time interval, the results of our study showed a small-scale pattern of variability. The CG shifted the spawning time from year to year: the first year it mostly spawned from 4:00 to 5:00 and with no spawning event from 2:00 to 3:00, while three years later the timing pattern was exactly the reverse. This gradual and annual spawning shift towards earlier hours can be attributed to intrinsic sources. Extrinsic sources were disregarded because we observed differences in the spawning time between the WG and the CG in 2009 and in 2010 despite the fact that both groups were exposed to the same environmental conditions, as their transport vessels sailed close to each other. We presume that the annual shift in the spawning hour displayed by the CG could be influenced by phenotypic traits such as body size or age. Consequently, we hypothesise that the annual differences in the CG might result from the annual growth of its individuals, which began below 75 kg in 2009 and reached 129 kg four years later. This hypothesis is supported by the coincidence in the spawning time of groups of similar individual size, the WG 2009 and 2010 and the CG 2012, all of similar individual weight and the same dominant spawning time (2:00–3:00 a.m.). This can be indicative that young spawners tend to spawn at later hours, shifting to earlier hours as they develop, but further research on a larger number of spawning groups is necessary to confirm this supposition. The literature on this topic, reproductive timing as a function of fish age, deals with timing variability within a season [41] but not within the day. However, those studies also showed that older fish spawn earlier in the season [41], [45]–[47]. The variability in the spawning time within a day has been observed and related with water temperature in other tuna species [35], [42]. These studies found that both species, yellowfin tuna and pacific bluefin tuna, tend to spawn in the evening at low temperatures, shifting towards the night at high temperatures. As these species have a wider spawning time, comprising hours with and without solar incidence, the observed shifts could be due to matching an optimal spawning water temperature. In the specific case of ABFT, time shifts were not temperature–related, and actually they cannot be expected since the extension of ABFT spawning time is too short and restricted to night-time. The sampling unit used in this study (55 minutes per station) prevented any inspection of spawning dynamics at shorter time units, but in-situ observations revealed that spawning events are highly synchronised, taking place in less than 15 minutes. Similarly, even shorter spawning events were observed in captive yellowfin tuna [35].

The onset of spawning took place between the end of May and the beginning of June and the differences between years were not temperature-related, varying between 19°C and 20°C. This range of temperature is considerably below what has been considered the threshold spawning temperature (24°C) for tuna [27] and closer to most recent estimations (20.5°C) [21]. It is worth mentioning that the onset of spawning was always close to the full moon around the end of May, which during the studied years shifted from the first week of June to the third week of May. In yellowfin tuna maximum egg production occurred mostly at greater illumination phases of the moon [35]. However, our time series is too short to infer the role of the lunar cycle or the role of the positive gradient of temperature in the onset of ABFT spawning. The spawning consistently slowed down in July and became negligible after the second week of July, a trend also inferred from ichthyoplankton studies [21], [48]. This result is consistent with the first dates of the post-spawning migration recently revealed by archival tags [49]. The peak of spawning was highly synchronized around the summer solstice, between June 15^th^ and 30^th^. Annual reproductive cycles with a well-defined periodicity are considered likely to be entrained to day length, as this is recognized to be the most reliable environmental signal of time [50]. Thus we hypothesized that day length, in particular the period of maximum day length, is the major factor of ABFT spawning synchronization. The period of the spawning peak is consistent with previous histological and immunohistochemical findings [16], [23] and takes place approximately 20 days after the first spawners are caught by the purse seine fleet. This time interval points towards a relatively extended period, around 3 weeks, between the arrival of spawners and the peak of spawning. Thus, the spawning period cannot be established merely from the presence of spawners in the spawning grounds. This was also depicted from the results of electronic tagging on ABFT spawners of the western population [29], and also from the eastern population [49]. In the eastern population was depicted a particular swimming behaviour associated with spawning activity during c.a 24 days but not during the whole period of the spawners' stay in the spawning grounds. Our results confirmed that this particular swimming behaviour is directly associated with the spawning activity, as it was only observed [49] during the spawning hours found in the present study and also extended for a similar number of days, c.a 21 days.

An unexpected result which might influence the reproductive success of ABFT was revealed by the profiles of daily temperature and cumulative spawning frequency curves. At first sight SST seems to play a major role in the temporal dynamics of spawning, but deeper examination revealed that the absolute SST value was unrelated with spawning disruption events. After storms the SST dropped but the temperatures reached were sufficient for spawning, as was proved in the preceding days. The SST fluctuations are indicators of oceanographic conditions, and their magnitude, negative or positive, indicates the strength of the unstable or stable conditions. We postulate that the annual reproductive success of ABFT might be influenced by the disruption of both oceanographic stability and water heating, rather than the absolute value of water temperature. Similarly, ellowfin tuna exhibited a long period of cessation at water temperatures sufficient for spawning but with a steadily decreasing trend [35].

This study presents the first results of *Thunnus thynnus* hatching time and proves the strong influence of temperature. Hatching time doubled from 26°C to 19.5°C (49 h-23 h), which is the range of temperatures observed in-situ from the beginning to the end of the spawning season. The inverse relationship of hatching time to water temperature would favour the synchronisation of larvae development, both the earliest and the latest spawners' progenies converging towards those of midterm. The influence of temperature on hatching time was higher than that theoretically expected for pelagic fishes [36] and for other tuna species [35], [51]. We might expect that ABFT eggs from early spawning would be exposed to planktonic and neustonic predators longer and their expectancy of survival would be lower than for eggs from later spawning. However, the spawning peak does not take place at the annual period of maximum water temperature but at the intermediate water temperatures of early summer, 22°–23°C, with maximum day length. Presumably, other factors from “match-mismatch” hypothesis [52], [53] to stabilizing selection [54] might have the reverse effect at both ends of the spawning period, balancing the offspring survival at both extremes.

The adaptive significance of the ABFT spawning period is unknown, but we dare say that in the Western Mediterranean its matching with the period of minimum night length might minimise predation on its early life stages. This hypothesis is based, firstly, on the distribution of ABFT eggs with higher abundance at the neuston than at lower water layers (Maria Santos, unpublished data), and secondly on the consideration that zooplankton predators might be more abundant at the surface during night hours as a result of the well-known nocturnal vertical migration. In Balearic waters the zooplankton distribution in the surface layer responds to the annual light cycle: descendent migration and arrival at the surface are synchronised with dawn and dusk, and depth reached at night followed as well the moonlight cycle [55], zooplankton reach shallower or deeper layers with new moon or full moon respectively. In particular, the abundance of *Pelagia noctiluca*, top planktonic predators [56], in the Balearic ABFT spawning ground is relevant and increasing [57]. The nocturnal abundance of *P. noctiluca* at the sea surface in this region [37] is indicative of the well-known pattern of diel vertical migration of this species [58]–[60]. A recent experimental study showed the voracity of *P. noctiluca* ephirae on ABFT eggs, being highly effective as a facultative neustonic predator [61]. Thus, we presume that spawning at the solstice might minimise the predation mortality of ABFT eggs.

Overall, this study shows the strength of the internal mechanism in ABFT that controls spawning traits. Spawning in ABFT is cyclical and highly synchronised on diel and annual scales. Thus we hypothesise that the timing of spawning is rather influenced by day length. In ectotermal animals, gametogenesis is expected to respond to the thermal regime during maturation [41], but Atlantic bluefin tuna are unique among teleosts fish for their endothermic capability, and the influence of temperature on spawning response could be weaker than in other species.
